# Intermittent Fasting to the Eye: A New Dimension Involved in Physiological and Pathological Changes

**DOI:** 10.3389/fmed.2022.867624

**Published:** 2022-05-24

**Authors:** Jiaqing Feng, Shijiao Zhang, Wenning Li, Tianle Bai, Yulin Liu, Xingyu Chang

**Affiliations:** The First Clinical Medical College, Lanzhou University, Lanzhou, China

**Keywords:** intermittent fasting, energy metabolism, eye, inflammatory response, eye diseases

## Abstract

Intermittent fasting (IF) is gaining popularity as a therapeutic dietary strategy that regulates metabolism and can alter the development of metabolic disorders. An increasing amount of research has connected ocular diseases to IF and discovered that it has a direct and indirect effect on the eye’s physiological structure and pathological alterations. This article summarizes the progress of research on IF in regulating the physiological structures of the ocular vasculature, the anterior segment of the eye, the retina, and the choroid. We explored the therapeutic potential of IF for various common ocular diseases. In the future, a comprehensive study into the fundamental processes of IF will provide a direct and rigorous approach to eye disease prevention and therapy.

## Introduction

Intermittent fasting (IF), also known as intermittent energy restriction (IER), is a new type of fasting therapy that alternates casual eating with restrictive eating for a set time, aiming to affect organism metabolism through dietary interventions. This easy-to-follow food restriction diet has been demonstrated to have numerous health benefits, including disease prevention and the slowing of aging. Dating back to 1997, Weindruch and Sohal showed that calorie restriction strategies might prolong lifespan by reducing body weight and oxidative stress (1). Subsequent studies have extended its beneficial effects to other aspects, such as diabetes, cardiovascular disease, cancer, and neurodegenerative diseases (2).

Currently, major IF diets include alternate-day fasting, periodic fasting, time-restricted fasting, and religion-related fasting. Alternate-day fasting is a form of fasting in which a day of normal eating alternates with a day of fasting, during which adequate water intake is ensured. It is currently the most common form of IF. Periodic fasting is a diet that involves fasting for 1 or 2 days per week and regularly eating the rest of the time. The 5:2 light fasting method is the most typical form of periodic fasting, where only 2 days of a week restrict dietary intake. During the time-limited fasting, participants fasted only at specific times of the day. Religion-related fasting is a prevalent form of IF based on religious beliefs. Ramadan fasting entails abstaining from all food and water from dawn to sunset for over a month, and abstaining from drugs, smoking, and sexual activity (3). Most eye-related population studies have been conducted around Ramadan fasting (4–7).

The eye is a vital sensory organ that receives most information from the external environment. With lifestyle changes, eye diseases and ocular complications of metabolic disorders, such as diabetes and hypertension, are consistently increasing. Chronic ocular diseases affect patients’ quality of life and impose a heavy economic burden on families and society. Dietary interventions have shown potential for prevention and adjunctive treatment in ocular pathologies due to its ease of acceptance and implementation. Nevertheless, there is a lack of evidence about IF and ocular disease. IF can alter a variety of ocular biological parameters and significantly impact some ocular surface diseases, retinopathies, etc. It is essential to understand the effects of IF on physical parameters and pathological changes in the eye. This review summarizes the benefits of IF on the eye and the prevention of ocular diseases by describing the effects of IF on various biological parameters of the eye and discusses possible physiological mechanisms of the effects of IF on the eye.

## Intermittent Fasting and Ocular Biological Parameters

**FIGURE 1:**
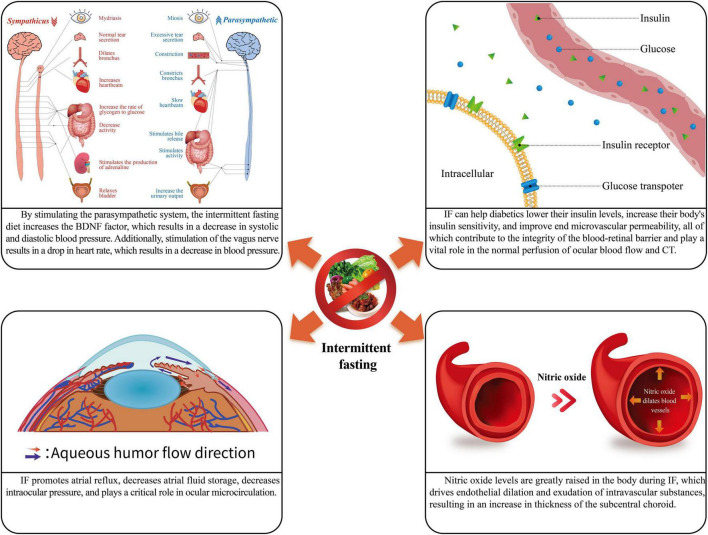
The physiological role of intermittent fasting. IF, intermittent fasting; CT, choroidal thickness.

As a new dietary pattern, IF has an important role in the body’s metabolism. At the same time, IF can also alter the sympathetic and parasympathetic tone and insulin sensitivity, improve blood circulation and flow patency, and thus alter ocular blood circulation, choroidal and retinal thickness, intraocular pressure (IOP), and the anterior segment of the eye. It has an important effect on the local metabolism and structure of the eye. This case is depicted in Figure 1 and Table 1.

**TABLE 1:** Effects of intermittent fasting on ocular biologic parameters.

Study title	Duration	Subjects	Projection	Results
***Eye microcirculation and blood pressure***				
Gokmen and Ozgur (25)	At least 10 days	Healthy male and female volunteers over the age of 18 with no known systemic disease, and who fasted regularly during Ramadan	Ramadan fasting. Healthy subjects were measured at the same time each day (1:30 P.M.–2:00 P.M.)	There was no significant difference in superficial and deep vascular density index between fasting and non-fasting period
Inan et al (15)	1 month	28 eyes of 28 normal subjects without ocular disease	Ramadan fasting after 14 h. The first measurements were performed in the fasting conditions and blood pressure, heart rate, and intraocular pressure were measured by color Doppler presentation after 1 month.	Non-fasting people had higher peak systolic velocity in the ophthalmic artery, central retinal artery, and temporal short posterior ciliary artery than fasting-healthy volunteers. The central retinal artery’s peak diastolic velocity was similarly higher in non-fasting people. Non-fasting individuals had a higher ophthalmic artery resistive index.
***Retinal and choroidal thickness***				
Uyar et al. (114)	1 month	A single eye of 87 healthy individuals	Ramadan fasting. Eyes were evaluated twice a day around 8.00 a.m. and 4.00 p.m. during Ramadan. Evaluations were repeated at the same time of the day, 1 month following Ramadan on the same subjects.	During fasting, temporal CT at 8 a.m. and foveal, temporal, and nasal CTs at 4 p.m. were significantly reduced. During fasting, the diurnal fluctuations in foveal and temporal CTs were significantly higher than the controls. In all segments measured at 4.00 p.m., retinal thicknesses were considerably decreased after fasting compared to controls.
Gokmen and Ozgur (25)	At least 10 days	Healthy male and female volunteers over the age of 18 with no known systemic disease, and who fasted regularly during Ramadan	Ramadan fasting. Healthy subjects were measured at the same time each day (1:30 P.M.–2:00 P.M.)	The choroidal thickness under the fovea center was found to be higher in the fasting period than in the non-fasting period. The mean total choroidal thickness was found to be reduced in the non-fasting period, although not to a statistically significant degree.
Ersan et al (30)	At least 20 days	42 healthy subjects with no ophthalmic or systemic disease	Ramadan fasting. Measured within 12 h after 21 consecutive days of fasting	CFT values were similar for fasting period and non-fasting period. The SFCT was significantly higher after consecutive fasting days toward the end of Ramadan, compared to the SFCT after 1 month of no fasting (1 month after Ramadan ended).
***Intraocular pressure***				
Kerimoglu et al. (42)	1 month	31 healthy subjects	Ramadan fasting. Measurements were taken at 0800 and 1600 h during Ramadan fasting and at 1 month during non-fasting periods.	Comparison of measurements between fasting and non-fasting periods at 0800 h revealed significantly higher values for IOP. Conversely at 1600 h, IOP was significantly lower during fasting.
Beyoğlu et al. (34)	ND	50 healthy fasting individuals in Ramadan (study group) and 50 healthy non-fasting subjects (control group)	Ramadan fasting. All measurement procedures are done between 16:00 and 17:00	There was a significant difference between Ramadan morning and control month morning. There was a significant difference between morning and afternoon of Ramadan. There was no significant difference between afternoon and dusk (after breaking one’s fast) in Ramadan.
Oltulu et al. (43)	ND	Seventy-two eyes of 72 fasting subjects (study group), and 62 eyes of 62 non-fasting subjects (control group)	Ramadan fasting. The participants of the control group were asked to consume daily meals of breakfast and lunch with adequate liquid on the day of ocular response analyzer (ORA) measurements. Measurements were taken between 17:00 and 18:00, approximately 14 h after the end of the fast	While fasting did not lead to any change in LD and CCT, it caused a small decrease in ACD and ACV, and a significant decrease in CD values.
Uysal et al. (44)	1 month	36 healthy fasting male subjects	Ramadan fasting. All measurements were recorded at 8:00 am and 4:00 p.m. during Ramadan and during a 1-month follow-up after Ramadan was over.	There was statistically significant difference within the two groups in IOPg and IOPcc.
***Preoptic biological parameters***				
Beyoğlu et al (34)	ND	50 healthy fasting individuals in Ramadan (study group) and 50 healthy non-fasting subjects (control group)	Ramadan fasting. All measurement procedures are done between 16:00 and 17:00	The difference of IOP between the two groups was statistically significant, and the IOP value decreased significantly
Oltulu et al. (43)	ND	Seventy-two eyes of 72 fasting subjects (study group), and 62 eyes of 62 non-fasting subjects (control group)	Ramadan fasting. The participants of the control group were asked to consume daily meals of breakfast and lunch with adequate liquid on the day of ocular response analyzer (ORA) measurements. Measurements were taken between 17:00 and 18:00, approximately 14 h after the end of the fast	CH and CRF significantly decreased in fasting periods compared with non-fasting periods.
Uysal et al. (44)	1 month	36 healthy fasting male subjects	Ramadan fasting. All measurements were recorded at 8:00 am and 4:00 p.m. during Ramadan and during a 1-month follow-up after Ramadan was over.	No difference in the ORA measurements including CH and CRF; CCT and CV values between fasting and non-fasting periods or within a single day (diurnal changes).

*ND, no data; CH, corneal hysteresis; CRF, corneal resistance factor; CT, choroidal thickness; CCT, central corneal thickness; ACD, anterior chamber depth; ACV, anterior chamber volume; CD, corneal density; LD, lens density; IOP-GAT, IOP with Goldmann applanation tonometer; IOPg, Goldman-correlated IOP; CFT, central foveal thickness; SFCT, subfoveal choroidal thickness; Ramadan fasting: after sunrise, no food and no water until sunset.*

### Intermittent Fasting and Ocular Blood Distribution

#### Intermittent Fasting Lowers Blood Pressure and Improves the Ocular Blood Supply

Hypertension plays a vital role in ocular blood perfusion and vascular distribution. The retina and other peripheral organs such as the brain and kidneys have similar vascular structures and physiological properties (8). When blood pressure rises, extensive retinal small artery stenosis and localized arteriovenous stenosis also occur, resulting in reduced retinal perfusion (9). If blood pressure remains high for an extended time, the blood-retinal barrier will be destroyed, leading to an exudative phase. Retinal hemorrhages and cotton wool spots appear during this period, leading to optic disc edema and macular edema (10). In addition, Hua et al. reported a significant decrease in vascular density in patients with a 5–10-year history of hypertension, a change which is likely related to the chronic effects of hypertension (11).

During early IF, systolic and diastolic blood pressure may be reduced without weight loss in obese men with prediabetes. This improvement in blood pressure is likely related to reducing insulin levels during fasting (12). It has been shown that both systolic and diastolic blood pressure decrease in hypertensive patients during prolonged IF (13, 14). Under the same conditions, there was no significant change in overall blood pressure between the fasting and non-fasting healthy groups. Still, peak systolic velocity in the ophthalmic artery, central retinal artery, and temporal short posterior ciliary artery were decreased by approximately 19.18, 43.60, and 13.92%, respectively (15). This decrease in blood pressure improves ocular vascular structure and density and maintains normal ocular perfusion (15).

**FIGURE 2:**
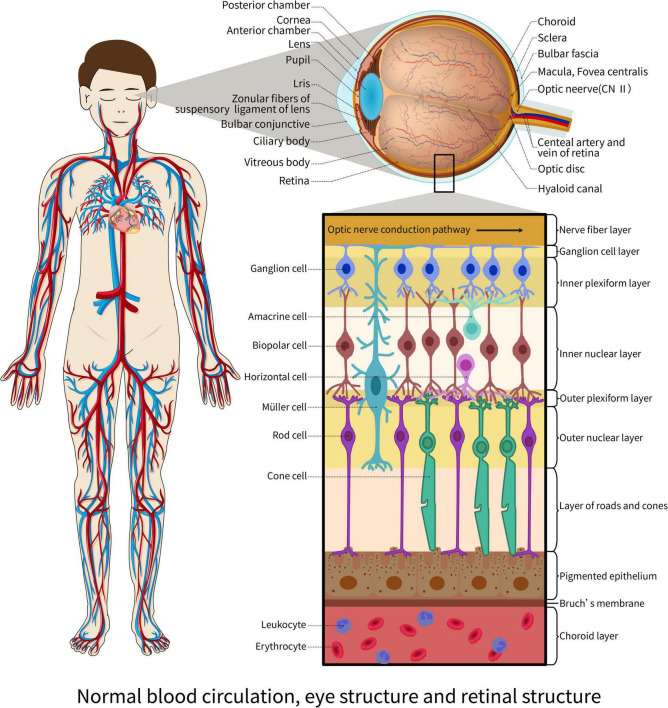
Normal blood circulation, eye structure, and retinal structure.

In addition, during IF, the sympathetic tone of the organism decreases, while the parasympathetic tone increases (16). Patients with hypertension have a problem with their autonomic function, which means their parasympathetic tone drops, and sympathetic nerves take over (17). This is because sympathetic nerves make immune cells produce proinflammatory factors, which send a signal to the brain that causes more sympathetic excitatory output (18, 19). On the other hand, sympathetic nerves can control how many hypertension-specific memory effector cells build up in the body (20). Under these effects, the blood vessels in the body are further constricted, and the blood pressure is further increased. In contrast, IF stimulates the vagus nerve, which innervates the heart and produces the opposite of sympathetic excitation, slowing cardiac conduction velocity, decreasing myocardial contractility, slowing heart rate, slowing heartbeat volume, and dilation of peripheral blood vessels, lowering blood pressure (17). Thus, IF can affect blood pressure by affecting sympathetic and parasympathetic tone affecting ocular blood flow. This case is depicted in Figure 2.

#### Intermittent Fasting Lowers Blood Glucose and Improves the Ocular Blood Supply

Chronic metabolic diseases such as diabetes can also affect blood flow to the eye. Normally, the avascular zone in the center of the foveal center is round or oval. This area is not bordered by gaps or breakdowns in the superficial or deep capillary plexus. In diabetic patients, both superficial and deep capillary plexuses show an increase in the size of the avascular zone in the central retinal notch (21). Multiple transcription factors are active in the early stages of diabetes. Increased pro-inflammatory chemicals in the retina trigger a low-grade inflammatory response that kills endothelial and pericytes, increases vascular permeability, disrupts the blood-retinal barrier, causes retinal edema, and worsens retinal ischemia over time (22). Chronic hyperglycemia can aggravate the condition by causing oxidative stress in the body and causing further damage to the microvascular endothelium (22).

Intermittent fasting can reduce insulin levels and increase the body’s sensitivity to insulin in healthy individuals (12). In the case of prediabetic and type 2 diabetic patients, IF can effectively reduce the glycated hemoglobin ratio, insulin levels, and insulin resistance (12). Through IF, it is possible to reduce blood glucose levels and improve end microvascular permeability in diabetic individuals, thus ensuring the integrity of the blood-retinal barrier and playing an essential role in the normal perfusion of ocular blood flow (23).

### Intermittent Fasting and Retinal and Choroidal Thickness

The retina is short for the optic part of the retina, a visual sensory organ that lies immediately within the inner layer of the choroid. The choroid is a highly vascularized tissue found between the retina and the sclera that gives appropriate oxygen and nutrients to the retina and is engaged in a range of retina-related functions (24).

An investigation has found no substantial changes in retinal thickness during IF (25), which might be due to a stringent self-regulatory system in the retina when perfusion pressures fluctuate dramatically (26, 27). Previously, animal studies found that retinal blood flow self-regulates in a similar manner (28). A similar phenomenon is observed in the human body (29). Unlike the retina, IF can cause choroid thickening (30).

The choroid’s self-regulation may cause a significant negative relationship between choroidal thickness and systolic blood pressure in the sub choroid (31, 32), and IF may thicken the choroid by lowering systolic blood pressure. In addition, there is a connection between neuromodulation and sub choroidal thickness. During IF, blood pressure drops, cardiac blood flow drops (33), and choroidal thickness under fovea increases when sympathetic tone drops and parasympathetic tone rises (16, 25). The choroidal thickness under the fovea increased by nearly 3% during the fasting period compared to the non-fasting period (25). Insulin sensitivity may influence the thickness of the choroidal layer at IF (34). There is a substantial thinning of choroidal thickness in diabetic patients and diabetic retinopathy (DR) patients (35). The nearly 37% reduction in choroidal thickness in the subcentral retinal recess compared to healthy subjects suggests that choroidal thickness is a great early predictor of DR. As a result, increased insulin sensitivity is expected to have a role in choroidal thickness change. Furthermore, nitric oxide levels in the body are greatly elevated during Ramadan (36). This stimulation of endothelial dilatation and intravascular material leakage may contribute to the increased choroidal thickness under the eye fossa (30).

### Intermittent Fasting and Intraocular Pressure

**FIGURE 3:**
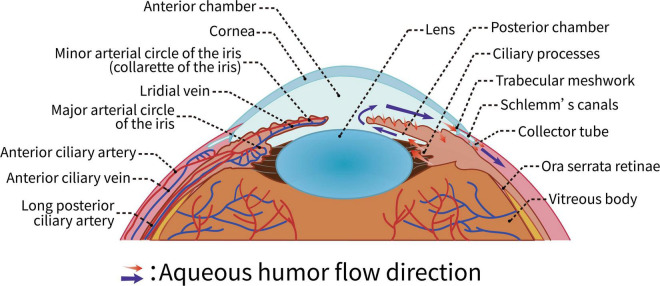
Aqueous humor circulation pathway.

The pressure created by the interplay of the eye’s contents on the eye’s wall and the contents of the eye is known as IOP. The intraocular contents include the lens, vitreous humor, and aqueous humor, of which the aqueous humor is the most critical factor affecting IOP. A balance between aqueous intake and outflow is required to maintain normal IOP. Any factor that interferes with the proper functioning of the aqueous humor circulation pathway may obstruct aqueous humor return and increase IOP. This case is depicted in Figure 3.

Previous studies have reached different conclusions. Some studies comparing the difference between IOP during fasting and non-fasting periods did not significantly change (11, 37). However, IF decreases insulin secretion and increases glucagon levels and sympathetic tone, which increases free fatty acid formation and thus increases local blood flow (38). Norepinephrine and cortisol produced during sympathetic excitation are elevated, and resistance to atrial outflow increases, causing IOP to rise. However, most studies have shown a significant decrease in IOP values during IF (34, 39–44). Kerimoglu et al. (42) found that in the Ramadan fasting population, IOP tended to be higher in the morning (fasting vs. non-fasting: 14.19 ± 3.53 mmHg vs. 12.03 ± 2.99 mmHg) and lower in the afternoon (fasting vs. non-fasting: 11.74 ± 2.39 mmHg vs. 13.13 ± 2.39 mmHg). This phenomenon may be related to dietary habits during Ramadan fasting (42). Fasting people intentionally consume more food and water before sunrise to tolerate hunger and thirst during the day (40), and in the morning, they drink more water, have an abundance of body fluids, and average aqueous humor production; in the afternoon, they become dehydrated because they have not drunk water for a long time, their plasma osmolality rises relatively, and the amount of aqueous humor obtained by ultrafiltration decreases relatively, with a disadvantage. Another study evaluated IOP during fasting in patients with open-angle glaucoma, and these patients showed an IOP decrease of approximately 26–32% at various times compared to the non-fasted state (45).

It has been shown that during IF, low-density lipoprotein (LDL) and apolipoprotein (Apo) B levels decrease, and low-density lipoprotein (HDL) and Apo A-1 levels increase significantly (46). The depletion of lipid stores during this period may reduce prostaglandin secretion, which leads to a decrease in IOP (45). In addition, IOP reduction may also be associated with increased body dehydration, neuro-endocrine system function, and inflammatory mediators in the uveoscleral channels (34).

Studies in recent years have favored IF to reduce IOP, and most of the periods measured were distributed across the period of IF with high reliability. However, further studies with more samples need to be selected to confirm the relationship between IOP and IF.

### Intermittent Fasting and Preoptic Biological Parameters

The anterior segment of the eye includes the anterior chamber, posterior chamber, lens suspensory ligament, atrial angle, conjunctiva, tear film, and other structures in front of the lens. Some parameters commonly used in clinical practice include anterior chamber depth, anterior chamber volume, central corneal thickness (CCT), corneal curvature, etc. These parameters play an essential role in the screening and diagnosis of ophthalmic diseases in clinical practice.

Intermittent fasting alters anterior chamber depth and volume, which is mostly due to dehydration This difference may be noticed in both fasting and non-fasting groups, and at various moments during the fasting period. Compared to the non-fasting group, anterior chamber depth and axial length were dramatically reduced in the fasted group (34, 47). Furthermore, during IF, the depth and volume of the anterior chamber were variable at different times of the day. According to Nowroozzadeh et al. (48), anterior chamber depth increased substantially during Ramadan, with nearly 26% compared to non-fasting periods. The anterior chamber depth values were greater in the morning than in the evening (*P* = 0.01), but the axial length of the eyes did not show such a significant change (48). Other studies with similar results have also been conducted (40). Furthermore, because there is a link between anterior chamber depth and IOP, with lower IOP being linked to deeper anterior chamber depth (48, 49), it is hypothesized that IF might change the magnitude of anterior chamber depth by affecting IOP.

In addition, Beyoğlu et al. (34) evaluated corneal density and lens density changes in IF During IF, subjects were found to have a decrease in corneal density (fasting vs. non-fasting: right/left eye: 12.81 ± 0.76/12.73 ± 0.73 vs. 13.28 ± 1.01/13.07 ± 0.77) (34), an expression likely due to changes in corneal inflammatory mediators and increased osmolarity as a result of dehydration (50, 51). However, lens density did not differ significantly between the fasting and non-fasting groups (34), probably because of lens microcirculatory water channels (52), which maintain a more stable density even during longer periods of dehydration.

The tear film is a crucial medium for maintaining lubrication between the cornea and the conjunctiva and keeping harmful substances such as bacteria from entering the eye. An aqueous layer, a lipid layer, and a mucin layer make up the typical tear film. Tear secretion levels were substantially lower during IF (42). The assessment of tear break-up time (TBUT) did not change significantly between fasting and non-fasting periods (50). In addition, the activity of several enzymes, such as lysozyme, lactoferrin, and alpha-amylase, was reduced throughout the fasting period in the fasted population (53).

## Intermittent Fasting and Eye Disease

### Intermittent Fasting and Diabetic Retinopathy

Diabetic retinopathy (DR) is a major cause of low vision and blindness in adults today (54, 55) and is one of the microvascular complications caused by diabetes. The primary pathogenic alterations are retinal ischemia, aberrant neovascularization, retinal inflammation, increased vascular permeability, and neuronal and glial abnormalities (56). The current treatment of DR is mainly retinal laser photocoagulation, vitreous cavity injection, and vitrectomy. However, it is still unable to manage the condition of DR (57), and this therapy is mostly used on patients with advanced DR, with early prevention focusing on food and lifestyle modifications to prevent the disease from progressing further.

#### Intermittent Fasting Suppresses the Diabetic Retinopathy Inflammatory Response

The inflammatory response is a crucial pathophysiology of DR. Inflammatory variables such as intraretinal IL-1β, TNF-α, and C-reactive protein are considerably enhanced in DR in previous studies (58). Further research discovered that exposing retinal endothelial cells (RECs) to the inflammatory cytokines IL-1β and TNF-α increased glucose consumption while also causing oxidative damage to the cells. High glucose levels promote the mobility of leukocytes and the activation of proinflammatory molecules, which increases the synthesis of VEGF and TGF-1 in RECs (59) and can cause direct damage to RECs (60). Furthermore, lipid dysregulation is involved in the inflammatory response in DR. REC dysregulation is an early indication of DR. Lowering cholesterol and ceramide levels in the endothelium can decrease the progression of DR (61).

By lowering blood glucose and cholesterol levels, decreasing the generation of late glycosylation final products and inflammatory cytokines, boosting the release of heat shock proteins and adiponectin, and speeding up cellular self-phagocytosis, IF can reduce the inflammatory response in DR (62, 63).

Sirtuin 1 (SIRT1) is a nutrient-sensing deacetylase that becomes activated in low-nutrient states. SIRT1 activation boosts insulin secretion, reduces insulin resistance and weight loss, and controls inflammation (64, 65). SIRT1 activation inhibits NF-κB, [ADP-ribose] polymerase (PARP-1), and matrix-metallopeptidase 9 (MMP-9) activation (66–68). It decreases histone acetylation of the DNA (cytosine-5)-methyltransferase 1 (DNMT1) promoter, preventing inflammation and mitochondrial damage. In contrast, in the diabetic retina, increased DNA methylation of the SIRT1 promoter leads to transcriptional repression (69). SIRT1 expression and activity are both reduced, leading to reduced LXR signaling and dysregulation of retinal cholesterol metabolism, as well as increased production of proinflammatory cytokines (69). IF increased Sirt1 mRNA expression in the liver and retina and significantly increased SIRT1 activity in the retina of mice compared to non-fasted animals. The increased deacetylase activity led to increased LXRα activity, and LXRα activation inhibited NF-κB-dependent proinflammatory gene upregulation in retinal cells (69). Thus, IF activates SIRT1/LXR signaling and provides vigorous support for the treatment of DR.

#### Intermittent Fasting Inhibits Abnormal Capillary Neogenesis in Diabetic Retinopathy

The destruction or decrease of pericytes, abnormal callogenesis, and disruption of the blood-retinal barrier are all important symptoms of early DR (70). Pericytes share a stromal sheath with the endothelial cells of retinal capillaries. Hypertension, hyperglycemia, the production of late glycation end products, and hypoxia may disrupt the barrier function between pericytes and endothelial cells, causing pericytes to apoptose and endothelial cells to generate additional capillaries owing to a lack of proliferative control (71). In DR, a reduction in the ratio of pericytes to capillary endothelial cells might lead to capillary degeneration and hence worsen hypoxia (72). Furthermore, the NF-κB pathway is activated, resulting in elevated levels of the intercellular adhesion factor ICAM-1 and vascular VCAM-1, which contribute to aberrant angiogenesis (73, 74). By reducing blood glucose and limiting the generation of proinflammatory molecules, IF can minimize retinal neovascularization and enhance retinal microcirculation (22, 56, 60). Based on the above, IF can be utilized as an additive force in treating DR that is anti-inflammatory and anti-vascularization in the near future.

#### Intermittent Fasting Improves Diabetic Retinopathy Glial Abnormalities

Müller cells, as the main glial cells of the retina, have a nutritional and supportive role in the retina (75). High glucose and hypoxic environments can lead to Müller cell inflammation and edema (70). Tu et al. (76) also showed an increase in oxidative stress and inflammatory responses in Müller cells in a diabetic model and decreased their protective paracrine factor secretion, which promotes retinal inflammation. Due to the lack of therapy options other than glycemic management, the prognosis of patients with DR is typically dismal before the onset of the proliferative phase (77). By enhancing insulin sensitivity and reducing blood glucose, IF can prevent aberrant alterations in Müller cells, implying that we may utilize it as an adjuvant treatment to prevent the beginning of DR, and postpone its development, give patients a favorable prognosis.

No specific treatment has been developed in the early stages of DR because there is no treatment other than glycemic control until the proliferative phase develops, therefore, patient prognosis is generally poor (77). IF prevents abnormal changes in Müller cells by improving insulin sensitivity and lowering blood glucose, which suggests that we can gain insight into the function of Müller cells and their role in the pathogenesis of DR and that using IF as an adjuvant therapy may prevent the onset of DR, delay the progression of DR, and provide a possibility for a good prognosis for patients.

### Intermittent Fasting and Dry Eye Syndrome and Ocular Surface Inflammation

The frequency and length of eye use have increased dramatically in modern society, as has the incidence of ocular surface illnesses. Dry eye is a multifactorial ocular surface disease defined by a tear film homeostasis problem, mainly associated with increased tear film instability, increased tear osmolarity and ocular surface inflammation development (78).

Diabetes is a systemic risk factor for dry eye, with more than half of type 2 diabetes patients experiencing dry eye symptoms (79). A sustained hyperglycemic condition damages the corneal epithelium directly, resulting in corneal ulcers and chronic corneal epithelial abnormalities (80). Hyperglycemia causes a decrease in conjunctival goblet cell density, a decrease in mucin density, and a decrease in tear film stability, in addition to direct epithelial damage; at the same time, hyperglycemia causes an increase in tear osmolarity, which causes mucin denaturation, a decrease in tear film stability, and yet another increase in osmolarity, creating a vicious cycle. A hyperglycemic environment can damage corneal neurons and impair neurotrophic function, which leads to the breakdown of corneal barrier integrity, decreased corneal sensitivity, decreased ocular surface gland production and blink frequency, and increased the advancement of dry eye (78, 81).

In diabetic patients, IF can effectively control blood glucose levels (82), resist the stress response, reduce autophagy, downregulate inflammatory expression (63), and reduce the damage caused by diabetes to the lacrimal vascular system and corneal autonomic nerves (80), resulting in corneal protection. On the other hand, a reduction in CD4 T cells has been observed to treat dry eyes in Sjogren’s syndrome, an autoimmune condition (83, 84). CD3, CD4 T cells, and CD19 B cells are all reduced by IF (85). This might potentially how IF helps with dry eye problems. In conclusion, IF may help diabetic individuals with dry eye symptoms and other ocular surface diseases.

However, IF might induce or worsen dry eye problems. Dehydration and decreased tear production can occur while fasting, resulting in dry eye symptoms. There are also substantial changes in tear composition, such as changes in tear film proteins and increased osmolarity (42, 50, 53), which can trigger the production of ocular surface proinflammatory cytokines, chemokines, and matrix metalloproteinases (MMPs) (8, 9, 30), thereby aggravating dry eye. As a result, the effects of IF on dry eye need to be investigated further, and its therapeutic benefits must be considered holistically.

### Intermittent Fasting and Glaucoma

Glaucoma, a progressive optic neuropathy defined by the loss of retinal ganglion cells, is the most prevalent neurodegenerative disease globally and results in permanent blindness (86). IOP that is pathologically raised is a significant risk factor for glaucoma. Glaucoma is caused by a disturbance of the aqueous circulation’s dynamic balance: a few cases are caused by excessive aqueous humor production, while the majority are caused by restricted aqueous outflows, such as constriction or even closure of the anterior chamber angle and trabecular sclerosis. Elevated IOP damages the visual nerve in two ways: mechanical compression and ischemia of the optic nerve. The greater the length of IOP increase, the more serious the visual function degradation (87). Current glaucoma therapy is still centered on reducing IOP. However, effectiveness is insufficient, and the condition might worsen even after the goal IOP is attained, which may be associated with progressive neuropathy.

By modifying the body’s metabolism, IF has been demonstrated to prevent aging and neurodegeneration (88, 89). Additionally, it can successfully reduce IOP and is critical in preventing the development of glaucoma. Although IF has been demonstrated to be advantageous in pathological neuropathy (90), there are minimal clinical data addressing retinal neurodegeneration, notably in glaucoma. Fasting for 48 h dramatically decreased acute IOP elevation-induced retinal ganglion cell loss, according to a recent study (86). The most plausible explanation for this phenomenon is that fasting regimens promote neuroprotection by inhibiting mTOR activity and activating cellular autophagy (91). Autophagy is the process of cytoplasmic breakdown and recycling *via* the autophagosomal-lysosomal pathway, which allows neurons to adapt to stressful environments and survive and is vital for neuronal homeostasis (91). Autophagic activity is inversely correlated with the activation state of the mammalian target of rapamycin (mTOR) and mTOR complex 1 (mTORC1) formation (92, 93). Activated mTOR inhibits the Unc51-like kinase 1 (ULK1) complex through phosphorylation, which inhibits autophagy (92, 93). In contrast, serine/threonine AMP-activated kinase (AMPK) is activated under low energy conditions and promotes autophagy by inhibiting mTORC1 and activating ULK1 (92, 94, 95). The study found a substantial drop in p-ULK1 in the fasting group of mice, indicating that IF can reduce the neurological damage caused by pathologically increased IOP in glaucoma by activating AMPK and thereby blocking mTOR signaling to promote autophagy.

At the same time, in EAAC1-deficient mice (a normal-tension glaucoma animal model), IF administered every other day for 7 weeks increased neurotrophic factor expression. It lowered oxidative stress levels, preventing retinal ganglion cell degeneration and improving visual impairment (91). Along with promoting neuronal cell survival, IF stimulates retinal glial cells and protects against damage induced by pro-inflammatory factor release. A calorie-restricted feeding regimen reduced ischemia-induced retinal damage and suppressed reactive gliosis in elderly rats (96).

The biochemical mechanisms underpinning the effects of IF on retinal nerve cells and glaucoma remain unknown. There are no conclusive clinical trials establishing a definite link between IF and glaucoma. Further research on the physiological effects of IF on the retina and genetic variables is critical for glaucoma and other ocular illness therapies.

### Intermittent Fasting and Other Eye Diseases

Other visual illnesses, such as autoimmune uveitis, age-related macular degeneration (AMD), and refractive error, may also be affected by IF. However, whether IF may definitively alter the onset and course of certain ocular illnesses is unknown.

Animals with provoked autoimmune uveitis had altered intestinal flora structure (97). As uveitis disease progressed, the experimental group of mice developed an increasingly distinct intestinal flora from controls, as well as an increase in the number of Treg cells in the retina, a decrease in cytokine levels, and the number of effector T cells in peripheral lymphoid tissues, and a decrease in the severity of uveitis following oral antibiotics. Further research (98) confirmed lymphocyte movement between the gut and the eye in uveitis, emphasizing the critical role of the intestinal flora composition in ocular illness. The above phenomenon may be explained by the concept of the intestinal-ocular axis, whereby the eye as a target organ is regulated by inflammatory factors and metabolites produced by flora in the intestine, such as TNF-α, bile acids, and SCFAs. Janowitz et al. (99) discovered that experimental autoimmune uveitis (EAU) mice exhibited lower intestinal α diversity than unimmunized animals before the beginning of ocular inflammation at the most severe stage of uveitis. The intestinal flora of EAU mice comprised Prevotella, Lactobacillus, Anaerobes, Parabacteroides, Firmicutes, and Clostridium, and an increase in the ileum. Intestinal microecology is a new aspect of the metabolic regulatory role of IF, and several studies have shown the potential of IF to alter flora composition. Pinto et al. (100) recently published a systematic review on the role of IF in remodeling the gut microbiota in humans and rats/mice. In this study, the authors found that both alternate-day fasting and time-restricted fasting regimens altered the ratio of intestinal flora, mainly the Firmicutes/Bacteroidetes ratio and Lactobacillus spp abundance. All these findings indicate that intestinal flora plays a role in the development and progression of autoimmune uveitis and that IF may impact disease progression by altering the makeup of the intestinal flora.

Age-related macular degeneration (AMD) is the leading cause of blindness in adults over 50 in developed countries (101) and ranks highly among ocular illnesses. Admas et al. investigated the impact of high and low glycemic index meals on the progression of AMD (102). Aged rats fed a high glycemic index diet developed retinal lesions like those associated with AMD, including hyperpigmentation, atrophy, lipofuscin deposition in the retinal pigment epithelium, and photoreceptor degeneration, whereas mice fed a low glycemic index diet did not. The AMD phenotype was reduced and even reversed in mice fed a high glycemic index diet. A low glycemic diet developed through IF may have the same impact on AMD prevention and therapy.

Refractive errors (RE), which encompasses myopia, hyperopia, astigmatism, refractive error, and presbyopia, is one of the most prevalent ocular illnesses affecting individuals of all ages (103). External parallel light traveling through the eye’s refractive system (the cornea, atrium, lens, and vitreous fluid) at rest does not concentrate on the central depression to generate a clear image. Previous research on IF and refractive error is limited, and the results are inconsistent. Some studies show (38) that Ramadan has no discernible effect on human vision or refractive error. However, several studies (38) discovered a modest difference in the CCT of fasting participants before and after Ramadan. In comparison, Gonen et al. (47) discovered that when fasting, the eye’s axial length was slightly shorter, the corneal thickness was thinner at night, indicating diurnal fluctuation, and the anterior chamber depth decreased throughout the night. Blurred vision during hyperglycemia has been the consequence of temporary refractive alterations produced by lens modifications (104), although it might also be caused by retinal abnormalities. The degree of retinal thickness is connected with visual acuity (105, 106), and can also cause changes in the length of the eye axis, which can result in refractive errors (104). As a result, we may speculate about the role of IF in altering changes in certain ocular biological parameters or reducing refractive errors in diabetic and hyperglycemic conditions through blood glucose control.

## Conclusion

**FIGURE 4:**
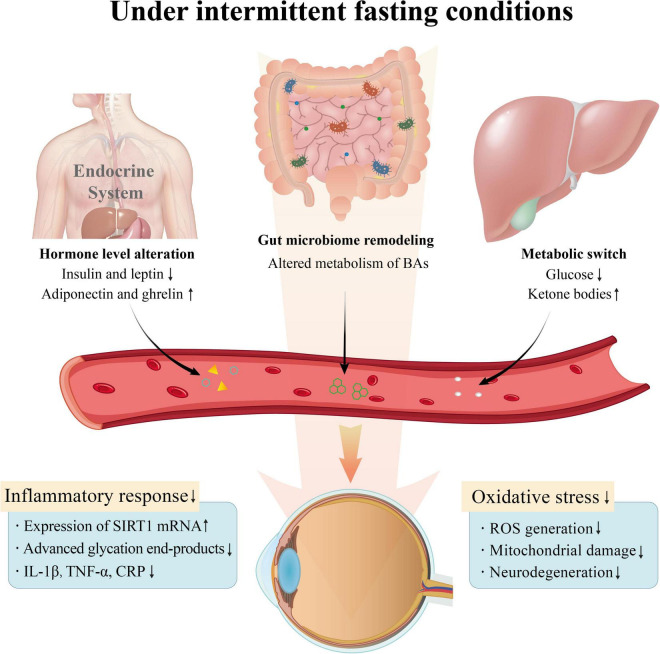
Effect of body metabolism on the eye under intermittent fasting.

Intermittent fasting improves the body’s metabolism and the local microenvironment of the eyes, hence preventing the development of some eye illnesses. IF affects the availability of nutrients. Limited nutrient intake drives the glucose-ketogenic metabolic switch in the body. Large amounts of endogenous ketone bodies replace glucose as an important energy source for the brain, muscles, eyes, and other organs (107). On the one hand, IF mitigates retinal neurovascular damage from sustained high glucose. On the other hand, ketone bodies serve as a better fuel to reduce ocular inflammation and oxidative stress (108, 109). Multiple metabolism-related hormone levels are upregulated in response to altered nutritional status, including increased ghrelin and adiponectin and decreased insulin and leptin (110). The secretion of these intestinal hormones regulates satiety, adapts to energy deficiency, and suppresses inflammation. Furthermore, this shift in eating patterns altered the body’s lipid metabolism, resulting in weight loss and decreased total cholesterol, triglycerides, and LDL cholesterol concentrations. On the other hand, IF increased cellular sensitivity to insulin, increased glucose uptake by cells in diabetic patients, decreased inflammatory factor production, inhibited neutrophil and other cell adhesion, and decreased platelet aggregation. In addition to its direct effect on nutrient intake, moderate hunger also influences the systemic and local stress state and inflammation levels. Fasting-mimicking therapy stimulated SIRT1/LXR signaling in retinal arteries and neurons in mice, resulting in lower expression of various inflammatory markers than controls (69). In summary, IF attenuates oxidative damage and the inflammatory response in the eye in multiple ways, which is common in primary and secondary eye damage. This case is depicted in Figure 4.

The favorable benefits of IF have also been demonstrated in the prevention of hypertension, where IF increased BDNF factors, activated the parasympathetic nervous system, decreased heart rate, and acted as a vasodilator on blood vessels, resulting in a drop in systolic and diastolic blood pressure. During IF, the body’s nitric oxide level increases dramatically, driving endothelial dilatation and intravascular material leakage, reducing blood pressure and alterations in choroidal thickness.

Intermittent fasting is used in various fields, including endocrine, cardiovascular, and tumor treatments, and it also has several beneficial effects on the body. However, this diet is not appropriate for everyone, particularly those who are underweight, pregnant, or nursing. Notably, the most immediate risk of IF is hypoglycemia in patients taking hypoglycemic medications, particularly insulin (both postprandial and basal) and sulfonylureas (including short-acting metronidazole), and caution should be exercised when administering IF to this group of patients (111). Intriguingly, IF regimens of different durations may have distinct or even opposite effects. Cerqueira et al. showed that a long-term calorie restriction strategy (32 weeks) induced glucose intolerance (112). Therefore, the selection of the period of IF protocol is a topic worthy of study.

Numerous investigations on the impact of IF on different ocular biological parameters have been conducted. However, research on ocular-related diseases such as glaucoma, retinopathy, and ocular surface diseases is still limited. Further research is needed to determine whether IF can enhance the local ocular environment and treat ocular diseases. The intestinal-ocular axis has a vital function in the eye, not only for gut bacteria but also for the makeup of ocular microorganisms, which play a critical role in ocular metabolism and illness. However, the existence of ocular surface microbiota remains a concern, and the effect of IF on ocular microorganisms is unknown. This may provide a new direction for IF study. Meanwhile, due to the lack of evidence for the advantages of IF alone and the low compliance of IF participants, most fasting therapies now employed in clinical practice are confined to diseases with established effectiveness, such as diabetes. If used for other ocular disorders, IF also searches for medications or chemicals that may be used in place of IF to lower calorie intakes, such as metformin, resveratrol, or rapamycin (113), or that can be used to complement in-hospital therapy with IF regimens. Thus, in addition to investigating changes in fundus parameters and IF for ophthalmology, we should also investigate changes in the body’s inflammatory response, metabolism, and gut flora and look for more effective therapies by integrating several therapeutic techniques.

## Author Contributions

JF and SZ contributed to the investigation and wrote the original draft of the manuscript. WL contributed significantly to the revision of this manuscript and the creation of the figures. TB and YL contributed significantly to the retrieval and screening of this manuscript. XC contributed to the methodology and conceptualization. All authors read and approved the final manuscript.

## Conflict of Interest

The authors declare that the research was conducted in the absence of any commercial or financial relationships that could be construed as a potential conflict of interest.

## Publisher’s Note

All claims expressed in this article are solely those of the authors and do not necessarily represent those of their affiliated organizations, or those of the publisher, the editors and the reviewers. Any product that may be evaluated in this article, or claim that may be made by its manufacturer, is not guaranteed or endorsed by the publisher.

## Abbreviations

IF: intermittent fasting
IER: intermittent energy restriction
FGF21: fibroblast growth factor 21
PGC-1 α: peroxisome proliferator-activated receptor γ coactivator 1 α
NAD: nicotinamide adenine dinucleotide
PARP1: polyadenosine diphosphate ribose polymerase 1
CD38: ADP-ribose cyclase
LEPR: leptin receptor
PRDM1: PR/SET structural domain 1
IGFBP-1: insulin-like growth factor binding protein-1
BDNF: brain-derived neurotrophic factor
HNF4 α: hepatocyte nuclear factor 4 α
ND: no data
CH: corneal hysteresis
CRF: corneal resistance factor
CT: choroidal thickness
CCT: central corneal thickness
ACD: anterior chamber depth
ACV: anterior chamber volume
CD: corneal density
LD: lens density
IOP: intraocular pressure
IOP-GAT: IOP with Goldmann applanation tonometer
IOPg: goldman-correlated IOP
CFT: central foveal thickness
SFCT: subfoveal choroidal thickness
LDL: low-density lipoprotein
HDL: high-density lipoprotein
Apo: apolipoprotein
DR: diabetic retinopathy
RECs: retinal endothelial cells
TUDCA: taurodeoxycholate
GPBAR1: G protein-coupled bile acid receptor 1
MMPs: matrix metalloproteinases
EAU: experimental autoimmune uveoretinitis
AMD: age-related macular degeneration (AMD).

## References

[1] Weindruch R Sohal RS. Seminars in medicine of the Beth Israel Deaconess Medical Center. Caloric intake and aging. *N Engl J Med.* (1997) 337:986–94. 10.1056/NEJM199710023371407 9309105 PMC2851235

[2] de Cabo R Mattson MP. Effects of intermittent fasting on health, aging, and disease. *N Engl J Med.* (2019) 381:2541–51. 10.1056/nejmra1905136 31881139

[3] Pietrocola F Pol J Vacchelli E Rao S Enot DP Baracco EE Caloric restriction mimetics enhance anticancer immunosurveillance. *Cancer Cell.* (2016) 30:147–60. 10.1016/j.ccell.2016.05.016 27411589 PMC5715805

[4] Brandhorst S. Fasting and fasting-mimicking diets for chemotherapy augmentation. *Geroscience.* (2021) 43:1201–16. 10.1007/s11357-020-00317-7 33410090 PMC8190229

[5] Goncalves MD Maddocks OD. Engineered diets to improve cancer outcomes. *Curr Opin Biotechnol.* (2021) 70:29–35. 10.1016/j.copbio.2020.10.007 33232844 PMC8702371

[6] Tourkmani AM Abdelhay O Alharbi TJ Bin Rsheed AM Azmi Hassali M Alrasheedy AA Impact of Ramadan-focused diabetes education on hypoglycemia risk and metabolic control for patients with type 2 diabetes mellitus: a systematic review. *Int J Clin Pract.* (2021) 75:e13817. 10.1111/ijcp.13817 33159361

[7] Liu Y Cheng A Li YJ Yang Y Kishimoto Y Zhang S SIRT3 mediates hippocampal synaptic adaptations to intermittent fasting and ameliorates deficits in APP mutant mice. *Nat Commun.* (2019) 10:1886. 10.1038/s41467-019-09897-1 31015456 PMC6478744

[8] Selvam S Kumar T Fruttiger M. Retinal vasculature development in health and disease. *Prog Retin Eye Res.* (2018) 63:1–19. 10.1016/j.preteyeres.2017.11.001 29129724

[9] Wong TY Mitchell P. Hypertensive retinopathy. *N Engl J Med.* (2004) 351:2310–7. 15564546 10.1056/NEJMra032865

[10] Lim HB Lee MW Park JH Kim K Jo YJ Kim JY. Changes in ganglion cell-inner plexiform layer thickness and retinal microvasculature in hypertension: an optical coherence tomography angiography study. *Am J Ophthalmol.* (2019) 199:167–76. 10.1016/j.ajo.2018.11.016 30502337

[11] Hua D Xu Y Zeng X Yang N Jiang M Zhang X Use of optical coherence tomography angiography for assessment of microvascular changes in the macula and optic nerve head in hypertensive patients without hypertensive retinopathy. *Microvasc Res.* (2020) 129:103969. 10.1016/j.mvr.2019.103969 31874131

[12] Lu Z Xie J Wu G Shen J Collins R Chen W Fasting selectively blocks development of acute lymphoblastic leukemia via leptin-receptor upregulation. *Nat Med.* (2017) 23:79–90. 10.1038/nm.4252 27941793 PMC6956990

[13] Grundler F Mesnage R Michalsen A Wilhelmi de Toledo F. Blood pressure changes in 1610 subjects with and without antihypertensive medication during long-term fasting. *J Am Heart Assoc.* (2020) 9:e018649. 10.1161/JAHA.120.018649 33222606 PMC7763762

[14] Wilkinson MJ Manoogian ENC Zadourian A Lo H Fakhouri S Shoghi A Ten-Hour Time-Restricted eating reduces weight, blood pressure, and atherogenic lipids in patients with metabolic syndrome. *Cell Metab.* (2020) 31:92–104.e5. 10.1016/j.cmet.2019.11.004 31813824 PMC6953486

[15] Inan UU Yücel A Ermis SS Oztürk F. The effect of dehydration and fasting on ocular blood flow. *J Glaucoma.* (2002) 11:411–5. 10.1097/00061198-200210000-00007 12362080

[16] Dong TA Sandesara PB Dhindsa DS Mehta A Arneson LC Dollar AL Intermittent fasting: a heart healthy dietary pattern? *Am J Med.* (2020) 133:901–7. 10.1016/j.amjmed.2020.03.030 32330491 PMC7415631

[17] Grassi G Ram VS. Evidence for a critical role of the sympathetic nervous system in hypertension. *J Am Soc Hypertens.* (2016) 10:457–66. 10.1016/j.jash.2016.02.015 27052349

[18] Biancardi VC Son SJ Ahmadi S Filosa JA Stern JE. Circulating angiotensin II gains access to the hypothalamus and brain stem during hypertension via breakdown of the blood-brain barrier. *Hypertension.* (2014) 63:572–9. 10.1161/HYPERTENSIONAHA.113.01743 24343120 PMC4080808

[19] Potapenko ES Biancardi VC Zhou Y Stern JE. Astrocytes modulate a postsynaptic NMDA-GABAA-receptor crosstalk in hypothalamic neurosecretory neurons. *J Neurosci.* (2013) 33:631–40. 10.1523/JNEUROSCI.3936-12.2013 23303942 PMC3756658

[20] Itani HA Xiao L Saleh MA Wu J Pilkinton MA Dale BL CD70 exacerbates blood pressure elevation and renal damage in response to repeated hypertensive stimuli. *Circ Res.* (2016) 118:1233–43. 10.1161/CIRCRESAHA.115.308111 26988069 PMC4833561

[21] Dimitrova G Chihara E Takahashi H Amano H Okazaki K. Quantitative retinal optical coherence tomography angiography in patients with diabetes without diabetic retinopathy. *Invest Ophthalmol Vis Sci.* (2017) 58:190–6. 10.1167/iovs.16-20531 28114579

[22] Semeraro F Morescalchi F Cancarini A Russo A Rezzola S Costagliola C. Diabetic retinopathy, a vascular and inflammatory disease: therapeutic implications. *Diabetes Metab.* (2019) 45:517–27. 10.1016/j.diabet.2019.04.002 31005756

[23] Semeraro F Cancarini A dell’Omo R Rezzola S Romano MR Costagliola C. Diabetic retinopathy: vascular and inflammatory disease. *J Diabetes Res.* (2015) 2015:582060. 10.1155/2015/582060 PMC4475523 26137497

[24] Balk LJ Oberwahrenbrock T Uitdehaag BM Petzold A. Physiological variation of retinal layer thickness is not caused by hydration: a randomised trial. *J Neurol Sci.* (2014) 344:88–93. 10.1016/j.jns.2014.06.031 25005893

[25] Gokmen O Ozgur G. The effect of religious fasting and dehydration at Ramadan on choroidal thickness and Retinal vessel densities, measured with optical coherence tomography angiography. *Eur J Ophthalmol.* (2021) 31:497–504. 10.1177/1120672120958297 32981332

[26] Kim SW Oh J Kwon SS Yoo J Huh K. Comparison of choroidal thickness among patients with healthy eyes, early age-related maculopathy, neovascular age-related macular degeneration, central serous chorioretinopathy, and polypoidal choroidal vasculopathy. *Retina.* (2011) 31:1904–11. 10.1097/IAE.0b013e31821801c5 21878855

[27] Delaey C Van De Voorde J. Regulatory mechanisms in the retinal and choroidal circulation. *Ophthalmic Res.* (2000) 32:249–56. 10.1159/000055622 11015035

[28] Zhang Y Wildsoet CF. RPE and choroid mechanisms underlying ocular growth and myopia. *Prog Mol Biol Transl Sci.* (2015) 134:221–40. 10.1016/bs.pmbts.2015.06.014 26310157 PMC4755498

[29] Feke GT Pasquale LR. Retinal blood flow response to posture change in glaucoma patients compared with healthy subjects. *Ophthalmology.* (2008) 115:246–52. 10.1016/j.ophtha.2007.04.055 17689612

[30] Ersan I Tufan HA Arikan S Kara S Gencer B Hondur AM. Effect of reduced meal frequency during ramadan fasting on retinal and choroidal thickness. *Semin Ophthalmol.* (2017) 32:418–21. 10.3109/08820538.2015.1118132 27082294

[31] Jung JW Chin HS Lee DH Yoon MH Kim NR. Changes in subfoveal choroidal thickness and choroidal extravascular density by spectral domain optical coherence tomography after haemodialysis: a pilot study. *Br J Ophthalmol.* (2014) 98:207–12. 10.1136/bjophthalmol-2013-303645 24187052

[32] Usui S Ikuno Y Akiba M Maruko I Sekiryu T Nishida K Circadian changes in subfoveal choroidal thickness and the relationship with circulatory factors in healthy subjects. *Invest Ophthalmol Vis Sci.* (2012) 53:2300–7. 10.1167/iovs.11-8383 22427554

[33] Turin TC Ahmed S Shommu NS Afzal AR Al Mamun M Qasqas M Ramadan fasting is not usually associated with the risk of cardiovascular events: a systematic review and meta-analysis. *J Family Community Med.* (2016) 23:73–81. 10.4103/2230-8229.181006 27186152 PMC4859102

[34] Beyoğlu A Karakucuk Y Çömez A. Evaluation of the effect of fasting on intraocular pressure, anterior segment parameters and density of crystalline lens and cornea. *Int Ophthalmol.* (2020) 40:2987–94. 10.1007/s10792-020-01482-6 32621018

[35] Esmaeelpour M Považay B Hermann B Hofer B Kajic V Hale SL Mapping choroidal and retinal thickness variation in type 2 diabetes using three-dimensional 1060-nm optical coherence tomography. *Invest Ophthalmol Vis Sci.* (2011) 52:5311–6. 10.1167/iovs.10-6875 21508108

[36] Yousefi B Faghfoori Z Samadi N Karami H Ahmadi Y Badalzadeh R The effects of Ramadan fasting on endothelial function in patients with cardiovascular diseases. *Eur J Clin Nutr.* (2014) 68:835–9. 10.1038/ejcn.2014.61 24755927

[37] Saraswathy S Tan JC Yu F Francis BA Hinton DR Weinreb RN Aqueous angiography: real-time and physiologic aqueous humor outflow imaging. *PLoS One.* (2016) 11:e0147176. 10.1371/journal.pone.0147176 26807586 PMC4725949

[38] Assadi M Akrami A Beikzadeh F Seyedabadi M Nabipour I Larijani B Impact of Ramadan fasting on intraocular pressure, visual acuity and refractive errors. *Singapore Med J.* (2011) 52:263–6. 21552787

[39] Nencioni A Caffa I Cortellino S Longo VD. Fasting and cancer: molecular mechanisms and clinical application. *Nat Rev Cancer.* (2018) 18:707–19. 10.1038/s41568-018-0061-0 30327499 PMC6938162

[40] Sarici AM Yuksel Elgin C Dikkaya F. Effect of fasting on corneal biomechanical and structural parameters. *Curr Eye Res.* (2016) 41:908–12. 10.3109/02713683.2015.1080279 26470948

[41] Sedaghat MR Heravian J Askarizadeh F Jabbarvand M Nematy M Rakhshandadi T Investigation of the effects of Islamic fasting on ocular parameters. *J Curr Ophthalmol.* (2017) 29:287–92. 10.1016/j.joco.2017.07.005 29270476 PMC5735232

[42] Kerimoglu H Ozturk B Gunduz K Bozkurt B Kamis U Okka M. Effect of altered eating habits and periods during Ramadan fasting on intraocular pressure, tear secretion, corneal and anterior chamber parameters. *Eye (Lond).* (2010) 24:97–100. 10.1038/eye.2009.96 19424289

[43] Oltulu R Satirtav G Ersan I Soylu E Okka M Zengin N. The effect of dehydration and fasting on corneal biomechanical properties and intraocular pressure. *Eye Contact Lens.* (2016) 42:392–4. 10.1097/ICL.0000000000000220 26657661

[44] Uysal BS Duru N Ozen U Arikan Yorgun M Akcay E Caglayan M Impact of dehydration and fasting on intraocular pressure and corneal biomechanics measured by the Ocular Response Analyzer. *Int Ophthalmol.* (2018) 38:451–7. 10.1007/s10792-017-0479-5 28255839

[45] Dadeya S Kamlesh, Shibal F Khurana C Khanna A. Effect of religious fasting on intra-ocular pressure. *Eye (Lond).* (2002) 16:463–5. 10.1038/sj.eye.6700089 12101455

[46] Antoni R Johnston KL Collins AL Robertson MD. Effects of intermittent fasting on glucose and lipid metabolism. *Proc Nutr Soc.* (2017) 76:361–8. 10.1017/S0029665116002986 28091348

[47] Baser G Cengiz H Uyar M Seker Un E. Diurnal alterations of refraction, anterior segment biometrics, and intraocular pressure in long-time dehydration due to religious fasting. *Semin Ophthalmol.* (2016) 31:499–504. 10.3109/08820538.2014.962179 25409203

[48] Nowroozzadeh MH Mirhosseini A Meshkibaf MH Roshannejad J. Effect of Ramadan fasting in tropical summer months on ocular refractive and biometric characteristics. *Clin Exp Optom.* (2012) 95:173–6. 10.1111/j.1444-0938.2011.00698.x 22248330

[49] Chakraborty R Read SA Collins MJ. Diurnal variations in axial length, choroidal thickness, intraocular pressure, and ocular biometrics. *Invest Ophthalmol Vis Sci.* (2011) 52:5121–9. 10.1167/iovs.11-7364 21571673

[50] Koktekir BE Bozkurt B Gonul S Gedik S Okudan S. Effect of religious fasting on tear osmolarity and ocular surface. *Eye Contact Lens.* (2014) 40:239–42. 10.1097/ICL.0000000000000044 24874298

[51] Meyer LM Kronschläger M Wegener AR. [Schleimpflug photography detects alterations in corneal density and thickness in patients with dry eye disease]. *Ophthalmologe.* (2014) 111:914–9. 10.1007/s00347-013-2964-1 25278346

[52] Mathias RT Kistler J Donaldson P. The lens circulation. *J Membr Biol.* (2007) 216:1–16. 10.1007/s00232-007-9019-y 17568975

[53] Sariri R Varasteh A Sajedi RH. Effect of Ramadan fasting on tear proteins. *Acta Medica (Hradec Kralove).* (2010) 53:147–51. 10.14712/18059694.2016.74 21171527

[54] Zhao K Liu J Dong G Xia H Wang P Xiao X Preliminary research on the effects and mechanisms of umbilical cord-derived mesenchymal stem cells in streptozotocin-induced diabetic retinopathy. *Int J Mol Med.* (2020) 46:849–58. 10.3892/ijmm.2020.4623 32626946

[55] Xiao F Li L Fu JS Hu YX Luo R. Regulation of the miR-19b-mediated SOCS6-JAK2/STAT3 pathway by lncRNA MEG3 is involved in high glucose-induced apoptosis in hRMECs. *Biosci Rep.* (2020) 40:BSR20194370. 10.1042/BSR20194370 32519748 PMC7327180

[56] Fehér J Taurone S Spoletini M Biro Z Varsányi B Scuderi G Ultrastructure of neurovascular changes in human diabetic retinopathy. *Int J Immunopathol Pharmacol.* (2018) 31:394632017748841. 10.1177/0394632017748841 29251013 PMC5849217

[57] Figueira J Fletcher E Massin P Silva R Bandello F Midena E Ranibizumab plus panretinal photocoagulation versus panretinal photocoagulation alone for high-risk proliferative diabetic retinopathy (PROTEUS Study). *Ophthalmology.* (2018) 125:691–700. 10.1016/j.ophtha.2017.12.008 29395119

[58] Kaštelan S Oreskovic I Bišćan F Kaštelan H Gverović Antunica A. Inflammatory and angiogenic biomarkers in diabetic retinopathy. *Biochem Med (Zagreb).* (2020) 30:030502. 10.11613/BM.2020.030502 32774120 PMC7394255

[59] McKinsey GL Lizama CO Keown-Lang AE Niu A Santander N Larpthaveesarp A A new genetic strategy for targeting microglia in development and disease. *Elife.* (2020) 9:e54590. 10.7554/eLife.54590 32573436 PMC7375817

[60] Liu J Chen S Biswas S Nagrani N Chu Y Chakrabarti S Glucose-induced oxidative stress and accelerated aging in endothelial cells are mediated by the depletion of mitochondrial SIRTs. *Physiol Rep.* (2020) 8:e14331. 10.14814/phy2.14331 32026628 PMC7002531

[61] Leal EC Aveleira CA Castilho AF Serra AM Baptista FI Hosoya K High glucose and oxidative/nitrosative stress conditions induce apoptosis in retinal endothelial cells by a caspase-independent pathway. *Exp Eye Res.* (2009) 88:983–91. 10.1016/j.exer.2008.12.010 19146853

[62] Golbidi S Daiber A Korac B Li H Essop MF Laher I. Health benefits of fasting and caloric restriction. *Curr Diab Rep.* (2017) 17:123. 10.1007/s11892-017-0951-7 29063418

[63] Patterson RE Sears DD. Metabolic effects of intermittent fasting. *Annu Rev Nutr.* (2017) 37:371–93. 28715993 10.1146/annurev-nutr-071816-064634 PMC13170603

[64] Wang RH Kim HS Xiao C Xu X Gavrilova O Deng CX. Hepatic Sirt1 deficiency in mice impairs mTorc2/Akt signaling and results in hyperglycemia, oxidative damage, and insulin resistance. *J Clin Invest.* (2011) 121:4477–90. 10.1172/JCI46243 21965330 PMC3204833

[65] Zabolotny JM Kim YB. Silencing insulin resistance through SIRT1. *Cell Metab.* (2007) 6:247–9. 10.1016/j.cmet.2007.09.004 17908551

[66] Kowluru RA Santos JM Zhong Q. Sirt1, a negative regulator of matrix metalloproteinase-9 in diabetic retinopathy. *Invest Ophthalmol Vis Sci.* (2014) 55:5653–60. 10.1167/iovs.14-14383 24894401 PMC4160094

[67] Kowluru RA Mishra M Kumar B. Diabetic retinopathy and transcriptional regulation of a small molecular weight G-Protein, Rac1. *Exp Eye Res.* (2016) 147:72–7. 10.1016/j.exer.2016.04.014 27109029 PMC4903942

[68] Mishra M Kowluru RA. Role of PARP-1 as a novel transcriptional regulator of MMP-9 in diabetic retinopathy. *Biochim Biophys Acta Mol Basis Dis.* (2017) 1863:1761–9. 10.1016/j.bbadis.2017.04.024 28478229 PMC5599300

[69] Hammer SS Vieira CP McFarland D Sandler M Levitsky Y Dorweiler TF Fasting and fasting-mimicking treatment activate SIRT1/LXRα and alleviate diabetes-induced systemic and microvascular dysfunction. *Diabetologia.* (2021) 64:1674–89. 10.1007/s00125-021-05431-5 33770194 PMC8236268

[70] Yumnamcha T Guerra M Singh LP Ibrahim AS. Metabolic dysregulation and neurovascular dysfunction in diabetic retinopathy. *Antioxidants (Basel).* (2020) 9:1244. 10.3390/antiox9121244 33302369 PMC7762582

[71] Beltramo E Porta M. Pericyte loss in diabetic retinopathy: mechanisms and consequences. *Curr Med Chem.* (2013) 20:3218–25. 10.2174/09298673113209990022 23745544

[72] Shi H Koronyo Y Fuchs DT Sheyn J Wawrowsky K Lahiri S Retinal capillary degeneration and blood-retinal barrier disruption in murine models of Alzheimer’s disease. *Acta Neuropathol Commun.* (2020) 8:202. 10.1186/s40478-020-01076-4 33228786 PMC7686701

[73] Song Y Tian X Wang X Feng H. Vascular protection of salicin on IL-1β-induced endothelial inflammatory response and damages in retinal endothelial cells. *Artif Cells Nanomed Biotechnol.* (2019) 47:1995–2002. 10.1080/21691401.2019.1608220 31106593

[74] Kinuthia UM Wolf A Langmann T. Microglia and inflammatory responses in diabetic retinopathy. *Front Immunol.* (2020) 11:564077. 10.3389/fimmu.2020.564077 33240260 PMC7681237

[75] Midena E Micera A Frizziero L Pilotto E Esposito G Bini S. Sub-threshold micropulse laser treatment reduces inflammatory biomarkers in aqueous humour of diabetic patients with macular edema. *Sci Rep.* (2019) 9:10034. 10.1038/s41598-019-46515-y 31296907 PMC6624368

[76] Tu Y Song E Wang Z Ji N Zhu L Wang K Melatonin attenuates oxidative stress and inflammation of Müller cells in diabetic retinopathy via activating the Sirt1 pathway. *Biomed Pharmacother.* (2021) 137:111274. 10.1016/j.biopha.2021.111274 33517190

[77] Robles-Rivera RR Castellanos-González JA Olvera-Montaño C Flores-Martin RA López-Contreras AK Arevalo-Simental DE Adjuvant therapies in diabetic retinopathy as an early approach to delay its progression: the importance of oxidative stress and inflammation. *Oxid Med Cell Longev.* (2020) 2020:3096470. 10.1155/2020/3096470 32256949 PMC7086452

[78] Craig JP Nichols KK Akpek EK Caffery B Dua HS Joo CK TFOS DEWS II definition and classification report. *Ocul Surf.* (2017) 15:276–83. 10.1016/j.jtos.2017.05.008 28736335

[79] Aljarousha M Badarudin NE Che Azemin MZ. Comparison of dry eye parameters between diabetics and non-diabetics in district of Kuantan, Pahang. *Malays J Med Sci.* (2016) 23:72–7. 27418872 PMC4934721

[80] Ljubimov AV. Diabetic complications in the cornea. *Vision Res.* (2017) 139:138–52. 10.1016/j.visres.2017.03.002 28404521 PMC5660664

[81] DeMill DL Hussain M Pop-Busui R Shtein RM. Ocular surface disease in patients with diabetic peripheral neuropathy. *Br J Ophthalmol.* (2016) 100:924–8. 10.1136/bjophthalmol-2015-307369 26500330 PMC5037046

[82] Vitale R Kim Y. The effects of intermittent fasting on glycemic control and body composition in adults with obesity and Type 2 diabetes: a systematic review. *Metab Syndr Relat Disord.* (2020) 18:450–61. 10.1089/met.2020.0048 32780629

[83] Trujillo-Vargas CM Schaefer L Alam J Pflugfelder SC Britton RA de Paiva CS. The gut-eye-lacrimal gland-microbiome axis in Sjögren Syndrome. *Ocul Surf.* (2020) 18:335–44. 10.1016/j.jtos.2019.10.006 31644955 PMC7124975

[84] Wang C Zaheer M Bian F Quach D Swennes AG Britton RA Sjögren-like lacrimal Keratoconjunctivitis in Germ-Free mice. *Int J Mol Sci.* (2018) 19:565. 10.3390/ijms19020565 29438346 PMC5855787

[85] Maifeld A Bartolomaeus H Löber U Avery EG Steckhan N Marko L Fasting alters the gut microbiome reducing blood pressure and body weight in metabolic syndrome patients. *Nat Commun.* (2021) 12:1970. 10.1038/s41467-021-22097-0 33785752 PMC8010079

[86] Russo R Nucci C Adornetto A. The promise of neuroprotection by dietary restriction in glaucoma. *Neural Regen Res.* (2022) 17:45–7. 10.4103/1673-5374.314308 34100425 PMC8451568

[87] Twa MD. Intraocular pressure and glaucoma. *Optom Vis Sci.* (2018) 95:83–5. 29389709 10.1097/OPX.0000000000001183

[88] Mattson MP. Energy intake and exercise as determinants of brain health and vulnerability to injury and disease. *Cell Metab.* (2012) 16:706–22. 10.1016/j.cmet.2012.08.012 23168220 PMC3518570

[89] Pani G. Neuroprotective effects of dietary restriction: evidence and mechanisms. *Semin Cell Dev Biol.* (2015) 40:106–14. 10.1016/j.semcdb.2015.03.004 25773162

[90] Gillette-Guyonnet S Secher M Vellas B. Nutrition and neurodegeneration: epidemiological evidence and challenges for future research. *Br J Clin Pharmacol.* (2013) 75:738–55. 10.1111/bcp.12058 23384081 PMC3575940

[91] Russo R Varano GP Adornetto A Nazio F Tettamanti G Girardello R Rapamycin and fasting sustain autophagy response activated by ischemia/reperfusion injury and promote retinal ganglion cell survival. *Cell Death Dis.* (2018) 9:981. 10.1038/s41419-018-1044-5 30250019 PMC6155349

[92] Kim J Kundu M Viollet B Guan KL. AMPK and mTOR regulate autophagy through direct phosphorylation of Ulk1. *Nat Cell Biol.* (2011) 13:132–41. 10.1038/ncb2152 21258367 PMC3987946

[93] Sengupta S Peterson TR Sabatini DM. Regulation of the mTOR complex 1 pathway by nutrients, growth factors, and stress. *Mol Cell.* (2010) 40:310–22. 10.1016/j.molcel.2010.09.026 20965424 PMC2993060

[94] Alers S Löffler AS Wesselborg S Stork B. Role of AMPK-mTOR-Ulk1/2 in the regulation of autophagy: cross talk, shortcuts, and feedbacks. *Mol Cell Biol.* (2012) 32:2–11. 10.1128/MCB.06159-11 22025673 PMC3255710

[95] Egan D Kim J Shaw RJ Guan KL. The autophagy initiating kinase ULK1 is regulated via opposing phosphorylation by AMPK and mTOR. *Autophagy.* (2011) 7:643–4. 10.4161/auto.7.6.15123 21460621 PMC3359466

[96] Kim KY Ju WK Neufeld AH. Neuronal susceptibility to damage: comparison of the retinas of young, old and old/caloric restricted rats before and after transient ischemia. *Neurobiol Aging.* (2004) 25:491–500. 10.1016/j.neurobiolaging.2003.07.005 15013570

[97] Nakamura YK Metea C Karstens L Asquith M Gruner H Moscibrocki C Gut microbial alterations associated with protection from autoimmune uveitis. *Invest Ophthalmol Vis Sci.* (2016) 57:3747–58. 10.1167/iovs.16-19733 27415793 PMC4960998

[98] Nakamura YK Janowitz C Metea C Asquith M Karstens L Rosenbaum JT Short chain fatty acids ameliorate immune-mediated uveitis partially by altering migration of lymphocytes from the intestine. *Sci Rep.* (2017) 7:11745. 10.1038/s41598-017-12163-3 28924192 PMC5603543

[99] Janowitz C Nakamura YK Metea C Gligor A Yu W Karstens L Disruption of intestinal homeostasis and intestinal microbiota during experimental autoimmune uveitis. *Invest Ophthalmol Vis Sci.* (2019) 60:420–9. 10.1167/iovs.18-24813 30695094 PMC6353239

[100] Pinto FCS Silva AAM Souza SL. Repercussions of intermittent fasting on the intestinal microbiota community and body composition: a systematic review. *Nutr Rev.* (2022) 80:613–28. 10.1093/nutrit/nuab108 35020929

[101] García-Layana A Cabrera-López F García-Arumi J Arias-Barquet L Ruiz-Moreno JM. Early and intermediate age-related macular degeneration: update and clinical review. *Clin Interv Aging.* (2017) 12:1579–87. 10.2147/CIA.S142685 29042759 PMC5633280

[102] Adams MK Simpson JA Aung KZ Makeyeva GA Giles GG English DR Abdominal obesity and age-related macular degeneration. *Am J Epidemiol.* (2011) 173:1246–55. 21422060 10.1093/aje/kwr005

[103] Sheeladevi S Seelam B Nukella PB Borah RR Ali R Keay L. Prevalence of refractive errors, uncorrected refractive error, and presbyopia in adults in India: a systematic review. *Indian J Ophthalmol.* (2019) 67:583–92. 10.4103/ijo.IJO_1235_18 31007213 PMC6498913

[104] Wiemer NG Eekhoff EM Simsek S Heine RJ Ringens PJ Polak BC The effect of acute hyperglycemia on retinal thickness and ocular refraction in healthy subjects. *Graefes Arch Clin Exp Ophthalmol.* (2008) 246:703–8. 10.1007/s00417-007-0729-8 18219490 PMC2292474

[105] Tian F Zheng D Zhang J Liu L Duan J Guo Y Choroidal and retinal thickness and axial eye elongation in Chinese junior students. *Invest Ophthalmol Vis Sci.* (2021) 62:26. 10.1167/iovs.62.9.26 34279570 PMC8297418

[106] Jin P Zou H Zhu J Xu X Jin J Chang TC Choroidal and retinal thickness in children with different refractive status measured by swept-source optical coherence tomography. *Am J Ophthalmol.* (2016) 168:164–76. 10.1016/j.ajo.2016.05.008 27189931

[107] Mattson MP Moehl K Ghena N Schmaedick M Cheng A. Intermittent metabolic switching, neuroplasticity and brain health. *Nat Rev Neurosci.* (2018) 19:63–80. 10.1038/nrn.2017.156 PMC5913738 29321682

[108] Cheung N Mitchell P Wong TY. Diabetic retinopathy. *Lancet.* (2010) 376:124–36. 20580421 10.1016/S0140-6736(09)62124-3

[109] Ryals RC Huang SJ Wafai D Bernert C Steele W Six M A Ketogenic & Low-Protein diet slows retinal degeneration in rd10 mice. *Transl Vis Sci Technol.* (2020) 9:18. 10.1167/tvst.9.11.18 PMC7571290 33117609

[110] Longo VD Mattson MP. Fasting: molecular mechanisms and clinical applications. *Cell Metab.* (2014) 19:181–92. 10.1016/j.cmet.2013.12.008 24440038 PMC3946160

[111] Corley BT Carroll RW Hall RM Weatherall M Parry-Strong A Krebs JD. Intermittent fasting in Type 2 diabetes mellitus and the risk of hypoglycaemia: a randomized controlled trial. *Diabet Med.* (2018) 35:588–94. 10.1111/dme.13595 29405359

[112] Cerqueira FM da Cunha FM Caldeira da Silva CC Chausse B Romano RL Garcia CC Long-term intermittent feeding, but not caloric restriction, leads to redox imbalance, insulin receptor nitration, and glucose intolerance. *Free Radic Biol Med.* (2011) 51:1454–60. 10.1016/j.freeradbiomed.2011.07.006 21816219

[113] Madeo F Carmona-Gutierrez D Hofer SJ Kroemer G. Caloric restriction mimetics against age-associated disease: targets, mechanisms, and therapeutic potential. *Cell Metab.* (2019) 29:592–610. 10.1016/j.cmet.2019.01.018 30840912

[114] Uyar E Dogan U Ulas F Celebi S. Effect of fasting on choroidal thickness and its diurnal variation. *Curr Eye Res.* (2019) 44:695–700. 10.1080/02713683.2019.1584677 30777786

